# Monitoring and Predictive Maintenance of Centrifugal Pumps Based on Smart Sensors

**DOI:** 10.3390/s22062106

**Published:** 2022-03-09

**Authors:** Lei Chen, Lijun Wei, Yu Wang, Junshuo Wang, Wenlong Li

**Affiliations:** School of Mechanical and Power Engineering, Zhengzhou University, No. 100 Science Street, Zhengzhou 450001, China; 202022202013978@gs.zzu.edu.cn (L.W.); wy1475899830@gs.zzu.edu.cn (Y.W.); wangyiwww123@163.com (J.W.); liwenlong2660@163.com (W.L.)

**Keywords:** centrifugal pump, smart sensor, edge computing, intelligent diagnosis, predictive maintenance

## Abstract

Centrifugal pumps have a wide range of applications in industrial and municipal water affairs. During the use of centrifugal pumps, failures such as bearing wear, blade damage, impeller imbalance, shaft misalignment, cavitation, water hammer, etc., often occur. It is of great importance to use smart sensors and digital Internet of Things (IoT) systems to monitor the real-time operating status of pumps and predict potential failures for achieving predictive maintenance of pumps and improving the intelligence level of machine health management. Firstly, the common fault forms of centrifugal pumps and the characteristics of vibration signals when a fault occurs are introduced. Secondly, the centrifugal pump monitoring IoT system is designed. The system is mainly composed of wireless sensors, wired sensors, data collectors, and cloud servers. Then, the microelectromechanical system (MEMS) chip is used to design a wireless vibration temperature integrated sensor, a wired vibration temperature integrated sensor, and a data collector to monitor the running state of the pump. The designed wireless sensor communicates with the server through Narrow Band Internet of Things (NB-IoT). The output of the wired sensor is connected to the data collector, and the designed collector can communicate with the server through 4G communication. Through cloud-side collaboration, real-time monitoring of the running status of centrifugal pumps and intelligent diagnosis of centrifugal pump faults are realized. Finally, on-site testing and application verification of the system was conducted. The test results show that the designed sensors and sensor application system can make good use of the centrifugal pump failure mechanism to automatically diagnose equipment failures. Moreover, the diagnostic accuracy rate is above 85% by using the method of wired sensor and collector. As a low-cost and easy-to-implement solution, wireless sensors can also monitor gradual failures well. The research on the sensors and pump monitoring system provides feasible methods and an effective means for the application of centrifugal pump health management and predictive maintenance.

## 1. Introduction

In the fields of industrial production and municipal water affairs, centrifugal pumps have a wide range of applications [1,2]. During the operation of centrifugal pumps, failures such as bearing wear and impeller imbalance often occur due to various factors such as equipment deterioration and environmental influences. These failures often occur with the increase in local temperature and the vibration of the pump [3]. By monitoring the physical quantities such as the vibration and temperature of the equipment, it is possible to perceive the operating status of the equipment, analyze and evaluate the health status of the centrifugal pump, and perform fault diagnosis and prediction for abnormal equipment to carry out predictive maintenance [4,5] work to ensure the safety of equipment and personnel [6].

Azadeh et al. [7] proposed a flexible algorithm to classify the condition of pumps based on support vector machine hyper-parameters optimization and artificial neural networks. Additionally, the result showed that the support vector classifier improves when it hybridizes with a genetic algorithm and particle swarm optimization. Orrù et al. [8] introduced a simple and easy machine learning model for early fault prediction of a centrifugal pump in the oil and gas industry. They validated the learning ability of machine learning models on the KNIME platform and potential faults are successfully recognized and classified ensuring good prediction accuracy. KNIME is an open-source software for creating data science. Gonçalves et al. [9] presented a novel output-only method based on the Markov parameters to diagnose faults. Additionally, the method was applied to diagnose incipient cavitation failures in a water supply network centrifugal pump. Ahmad et al. [10] proposed a fault diagnosis method for multistage centrifugal pumps using informative ratio principal component analysis. These studies mainly focus on the feature extraction of the collected state data, to achieve the purpose of classifying equipment failures. The quality of the state data often determines the effect of the post-classification algorithm, which cannot be ignored. In addition, the collection quality of equipment operating status information directly affects whether the actual project can be successfully implemented.

Given the above situation, this paper focuses on the research and design of intelligent collection equipment for centrifugal pump operating status information, and further proposes an IoT system architecture for centrifugal pump status monitoring and fault diagnosis, to realize real-time monitoring, health evaluation, fault diagnosis, and operation trend prediction of pump equipment.

The following sections are structured as follows: Section 2 analyzes the application scenarios of this design; Section 3 presents the details of this design. Section 4 further introduces the proposed general framework. Section 5 presents the experiments and analysis of the results. Finally, in Section 6, conclusions and future research are described.

## 2. Centrifugal Pump Operation Failure and Its Features

**Figure 1:**
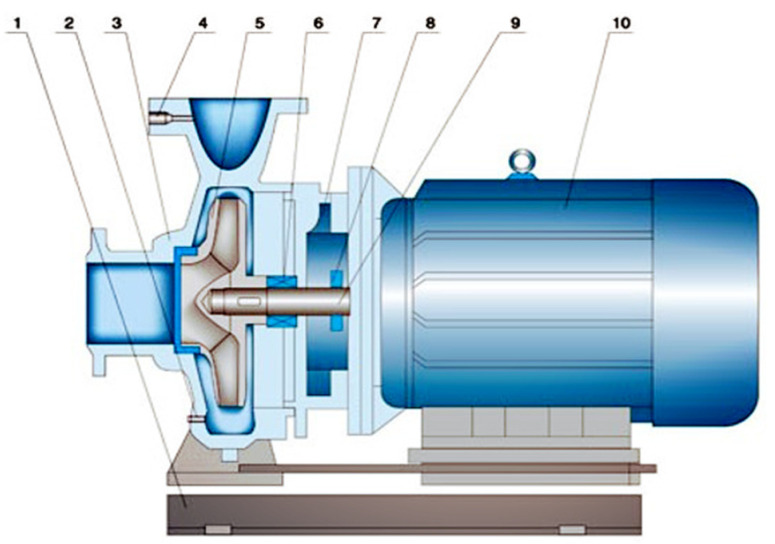
Typical structure diagram of the centrifugal pump unit. 1. Base. 2. Waterproof port. 3. Pump body. 4. Pressure tap. 5. Impeller. 6. Bearing. 7. Support frame. 8. Water retaining ring. 9. Pump shaft. 10. Motor.

The structure of a common single-stage horizontal centrifugal pump is shown in Figure 1. It is mainly composed of the base, bearing, pump body, pump cover, pump shaft, impeller, sealing ring, water retaining ring, and stuffing box [11,12,13].

After the centrifugal pump is switched on, the pump shaft will drive the impeller to rotate at a high speed, forcing the pre-filled liquid between the blades to rotate. Under the action of inertial centrifugal force, the liquid moves radially from the center of the impeller to the outer periphery [14]. At the same time as the outer circumference, a low-pressure area will be formed in the center of the impeller, causing the liquid to be sucked into the center of the impeller. Relying on the continuous operation of the impeller, the liquid is continuously sucked and discharged. Due to the structure and working principle of the centrifugal pump, as the equipment ages or the fluid is abnormal, some mechanical and fluid failures will occur. These failure forms are shown in Table 1, mainly including loose fault, imbalance fault, misalignment fault, bearing fault, cavitation, water hammer, abnormal flow passage, etc. [1,2,12,15]. These failure forms will cause changes in the pump vibration. Therefore, a sensor and IoT system [16,17,18,19,20] can be designed to form a digital unit and an industrial internet platform for smart pumps, which can effectively perform signal acquisition, analysis, feature extraction, and fault diagnosis, and realize the digital upgrading of pump equipment and the intelligent operation and maintenance of pump units [21,22].

### 2.1. Mechanical Failure

#### 2.1.1. Bearing Fault and Feature

The bearing is an important part of the centrifugal pump that supports the rotor. Due to poor lubrication, overload, and other reasons, bearing failures will occur. Pitting, peeling, wear, and other features appear on the rolling bearing components, causing bearing wear, vibration, and impact [1,2,23]. As the wear continues and intensifies, it is often accompanied by a rise in temperature. It can be monitored and judged by the total vibration value, the vibration value under the narrow band frequency, and the kurtosis index reflecting the impact characteristics [24].

#### 2.1.2. Misalignment Fault and Feature

If there is a displacement or angular deviation of the shaft centerline at both ends of the centrifugal pump coupling, it will cause misalignment and increase vibration. In terms of frequency spectrum characteristics, it often causes changes in vibration amplitude at two times the operating frequency [25]. Depending on the cause of the misalignment, angular deviation, or displacement deviation, which causes vibration changes in the axial, radial, or two directions at the same time, it can be monitored and judged by the data of the two axial directions of the three-axis sensor.

#### 2.1.3. Imbalance Fault and Feature

Due to processing or assembly errors, uneven material quality distribution, or impeller defects, fouling or blockages during operation, the rotating parts of the centrifugal pump have eccentric or unbalanced masses, causing unbalanced forces. Under the excitation of unbalanced force, the vibration response of the system changes [26]. The vibration change caused by imbalance is mainly manifested at the power frequency and the vibration amplitude changes with the change in the speed.

#### 2.1.4. Loose Fault and Feature

Centrifugal pump loosening failure generally manifests as two types of loose foundation or looseness caused by the poor fit between components [27,28]. Among them, loose foundation, such as loosening of foundation screws, has the characteristics of prominent working frequency components and fixed vibration direction in vibration characteristics; looseness caused by poor assembly often has spectral characteristics of superimposed working frequency and high-order harmonics.

### 2.2. Fluid Fault

#### 2.2.1. Abnormal Flow Passage and Feature

During the operation of the centrifugal pump, due to blockages in the volute, improper assembly of the impeller, etc., the overall vibration of the centrifugal pump will increase and the pump efficiency will be reduced [13]. The characteristics of the vibration signal are mainly manifested as the blade passing frequency in the vibration signal is outstanding and the vibration intensity increases significantly with the increase in the rotation speed of the centrifugal pump.

#### 2.2.2. Water Hammer Fault and Feature

Due to the sudden opening and shutdown of the centrifugal pump or the sudden change in the valve, the phenomenon of shock caused by the sudden change in the flow in the pump body and the surrounding pipeline is called a water hammer [29]. A slight water hammer is often accompanied by a short and weak vibration and noise. A severe water hammer can make the pressure in the pump body or pipeline hundreds of times higher than normal, causing damage to the centrifugal pump components or pipeline bursts, etc. [30]. When a centrifugal pump water hammer failure occurs, it is manifested as the amplitude of the time-domain waveform of the vibration signal increasing sharply and then decreasing rapidly. It is a typical shock signal, and the amplitude of the high-frequency part is more prominent in the frequency domain.

#### 2.2.3. Cavitation Fault and Feature

Cavitation is a common problem of centrifugal pumps, which can cause an increase in pump vibration and noise, decrease in performance, and cause serious damage to pump parts [31,32]. There are many reasons for cavitation, but from the perspective of vibration response, whether it is caused by turbulence, internal reflux, or other reasons, it will show a shock vibration response [33]. The vibration feature of such a cavitation fault is a continuous wide-band signal. At the bottom of the spectrogram, there will be an overall uplift relative to the normal signal. Generally, there is a response from 300 Hz (or even lower) to the upper limit of the frequency band. A band-pass filter can be added to the signal, and for a signal in the passband, such as 500–2000 Hz, features are extracted to determine whether cavitation occurs or not.

## 3. Smart Collection Equipment

**Figure 2:**
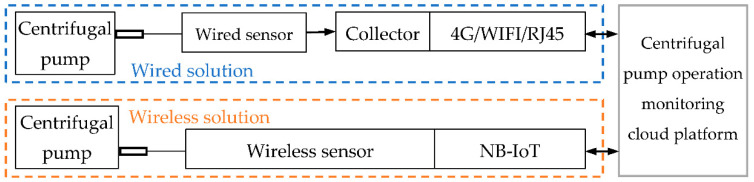
System topology diagram of the smart collection equipment.

To realize the purpose of intelligent diagnosis of the centrifugal pump, the data acquisition equipment installed on the centrifugal pump is designed and developed to provide a data basis for the realization of the system functions, mainly including wireless sensors, wired sensors, and intelligent collectors. The wireless sensor is connected with the cloud platform data layer interface through NB-IoT [34,35] to form a wireless application scheme; the wired sensor result is connected to the data collector, and the data collector uploads the collected result to the cloud server through 4G communication to form a wired application. Solution: according to the different application scenarios of the monitored pump, wireless or wired sensors can be used to complete data collection. The system topology diagram of the smart collection equipment is shown in Figure 2.

### 3.1. Wireless Sensor

**Figure 3:**
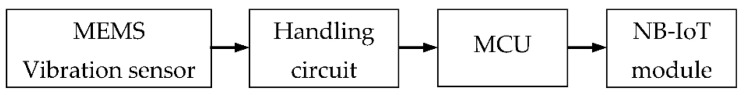
Block diagram of a wireless sensor circuit.

The wireless sensor transmits data to the server through the NB-IoT communication method. However, because of the battery power supply, the collection and transmission need to consume electrical power, which affects its battery life. From the perspective of industrial equipment maintenance, the sensor needs to have a service life of 2 years or more, and then the battery is replaced during the maintenance process. Data transmission adopts an interval collection and transmission method, and a group of data is transmitted every half an hour under rated conditions to ensure the service life of the battery. The sensor adopts MEMS chip design and integrates three-axis vibration and 1-channel temperature measurement to obtain a more comprehensive source of information. It supports MQTT (Message Queuing Telemetry Transport) data communication protocol, and the edge calculation function supports digital integration, vibration characteristic value, statistical value calculation functions, and can determine whether to transmit the complete original signal transmission schedule according to whether the set feature value threshold exceeds the standard. The parameters used to configure the collector can be issued from the server, and OTA (Over The Air) remote upgrade is supported. The circuit block diagram of the wireless sensor is shown in Figure 3.

Since the wireless sensor is battery-powered and needs to collect and transmit data at intervals, this working mechanism makes the diagnostic function of the wireless sensor solution limited. Some occasional equipment failures cannot be effectively monitored and judged, such as water hammer failures of centrifugal pumps, but for the gradual deterioration of equipment failures, such as imbalance, bearing wear, etc., it can still be effectively monitored and diagnosed.

### 3.2. Wired Sensors and Collectors

**Figure 4:**
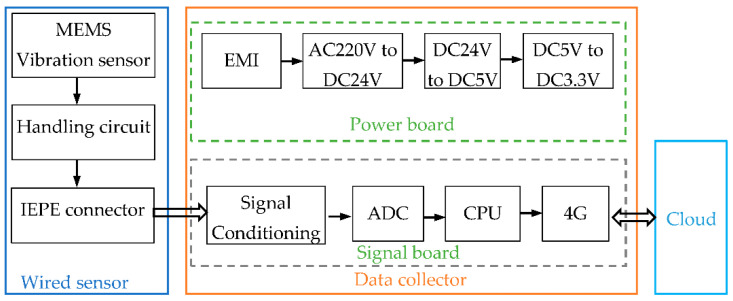
Block diagram of wired sensor and collector circuit principle.

The wired sensor is connected to the collector through the IEPE (Integrated Electronics Piezo-Electric) interface, the analog quantity is converted into a digital quantity through the collector to complete the digitization and carry out the edge calculation to realize the signal feature extraction. Due to the external power supply operating mode, data acquisition and processing can be performed continuously and in real time. The block diagram of the wired sensor and collector circuit principle is shown in Figure 4. The wired sensor uses the same triaxial MEMS sensor chip as the wireless sensor to ensure the consistency of bandwidth and measurement accuracy when the wireless and wired solutions collect vibration signals. The analog output of the wireless sensor is connected to the collector through a standard interface. The collector uses 220 V AC power supply, has 4G, WIFI, RJ45, RS485, and other data interfaces, and has rich data communication functions. A 24-bit AD converter is adopted to ensure the measurement accuracy requirements. The collector processor is designed with a high-performance ARM (Advanced RISC Machine) processor and is equipped with an embedded LINUX operating system to ensure rich edge data preprocessing capabilities. The embedded program supports OTA remote upgrades. In addition to retaining the vibration measurement interface, the collector can also be connected to process quantity sensor signals such as pressure and flow.

Wired sensors can realize continuous data collection and embedded software can easily realize the functions of signal preprocessing, filtering, integration, feature value calculation, and real-time fault judgment according to the configuration of the server and the threshold setting. Combined with feature judgment, it can also effectively monitor occasional faults.

### 3.3. Monitoring and Diagnosis

The embedded programs of wireless sensors and collectors integrate the basic algorithm library, which can perform calculations such as filtering, integration, Fourier transform, etc., extract the main vibration characteristic index, and then determine whether to upload complete raw data according to the magnitude of the feature indicators, reduce the requirements of network transmission bandwidth, and increase the real-time monitoring and diagnosis of equipment failures.

Digital devices such as wireless sensors and collectors upload the characteristic parameters of vibration and part of the raw data to the server. The server software conducts data modeling and analysis and divides into a variety of working conditions according to the different operating loads and speeds of centrifugal pumps, and finally completes the automatic identification and diagnosis of common faults to realize the intelligent operation and maintenance of equipment.

## 4. Overall System Architecture

**Figure 5:**
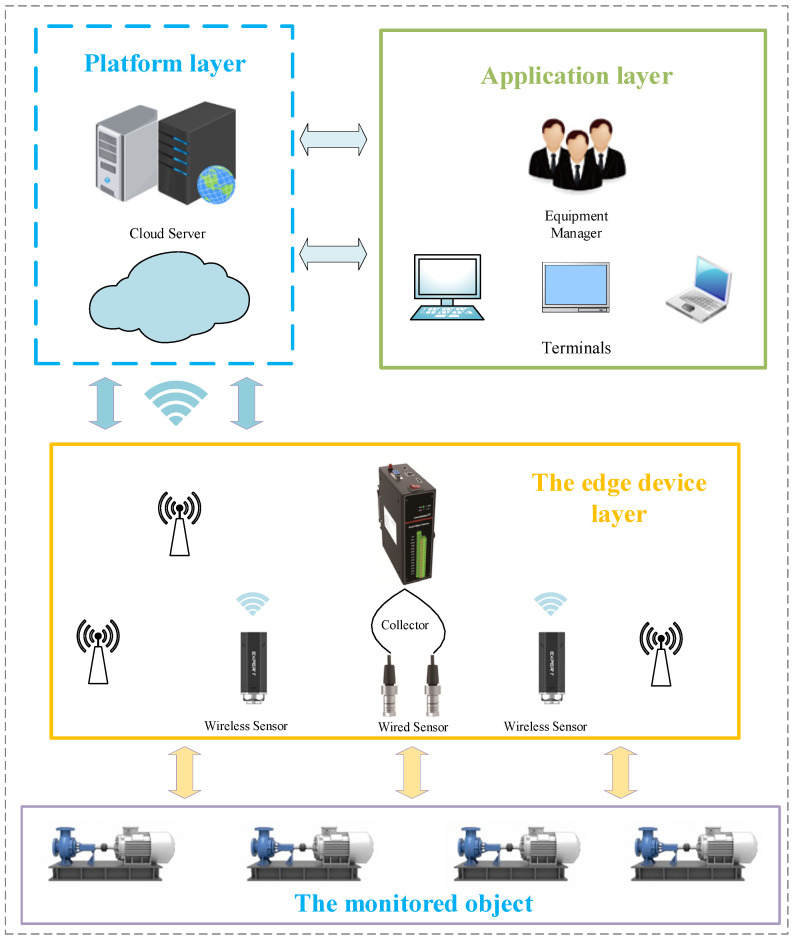
System topology diagram.

For better application in reality, we further propose an IoT system architecture for centrifugal pump status monitoring and fault diagnosis in this paper. The system topology diagram is shown in Figure 5. It is mainly composed of four main parts: the monitored object, the edge device layer, the platform layer, and application layer.

The first layer is the monitored object, that is, the equipment we need to monitor. In this article, it is the centrifugal pump unit.

The second layer is the edge device layer, which mainly includes sensors and collectors. Through these sensors and collectors, the digitization of pump equipment operation information is realized. The digitized equipment is an important part of the pump and completes the intelligent upgrade of the pump. In the edge collection equipment, complete signal collection, and preprocessing, the edge calculation and judgment of main features are realized and cooperate with the data model of the cloud platform to realize edge–cloud collaboration and improve the real-time and reliability of monitoring and diagnosis.

The third layer is the platform layer, which mainly provides an Internet service platform, consisting of a cloud server and software running on it. Data modeling and algorithm improvement can be performed on the server. The operation status evaluation and fault diagnosis of the equipment are realized according to the data collected by the edge device layer. With the continuous accumulation of data, the platform layer can realize the big data learning of alarm thresholds and send the learning results and judgment criteria to the edge hardware devices. Finally, the analysis results of the cloud server are provided to end-users through the application layer.

The last layer is the application layer. The cloud server provides algorithms and computing power for the monitoring, evaluation, diagnosis, and prediction of centrifugal pumps, and connects the diagnosis results with enterprise business systems to form a centrifugal pump health management and predictive maintenance system. Equipment managers can check the running status of monitored objects on various devices such as web pages and mobile terminals and arrange maintenance and production plans in a targeted manner according to the results of monitoring and diagnosis.

## 5. Experimental Testing and Application Verification

### 5.1. Test Environment

**Figure 6:**
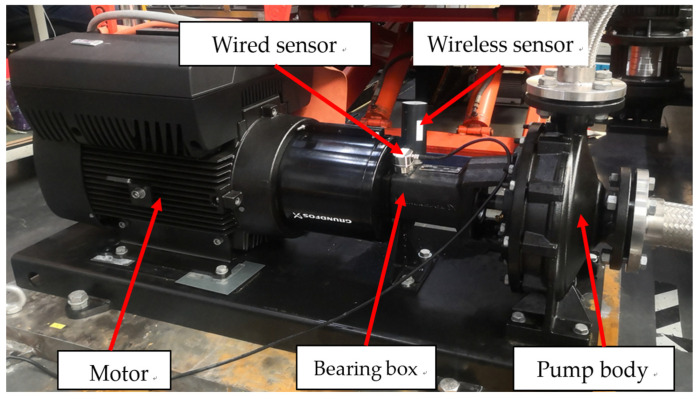
Experimental setup for fault diagnosis of the centrifugal pump.

To verify the availability of the designed sensors in the pump intelligent diagnostic system, the centrifugal pump produced by Grundfos was selected, which has a typical pump structure. A test bench was built on this basis, as shown in Figure 6.

The wireless sensor and the wired sensor were installed vertically side by side on the bearing box of the pump. The data collection was carried out by wireless sensor, wired sensor, and collectors, respectively, and the intelligent diagnostic test verification of centrifugal pump imbalance, misalignment, looseness, cavitation, bearing wear, and other faults was completed. The vibration diagnostic experiment is carried out on centrifugal pumps, and the experimental centrifugal pump includes NK, NKE, CR, etc. Grundfos series centrifugal pump. In this article, the diagnosis of centrifugal pump fault analysis is described as an example of experiments on the NKE pump. The main parameters of the experimental pump are shown in Table 2. Experiments are carried out with independently developed wireless and wired three-axis acceleration sensors. These sensors have a sensitivity of 200 mV/g. These sensors are fastened in the radial direction of the bearing housing using strong glue bonding.

### 5.2. The Process of Testing

To more realistically simulate the various failures of centrifugal pumps in reality, we conducted different experiments. Detailed test steps for different kinds of faults are shown below:

(1) Imbalance fault:

Make 3 impellers with different unbalance levels in the factory, the unbalance levels are 11, 15, and 20 g.

Step 1: Install the unbalance impeller on the pump.

Step 2: Set the pump speed to 1800, 2400, or 3000 r/min (almost 60%, 80%, 100% of the rated speed).

Step 3: Power on the pump, and keep the pump working for 5–10 min.

Step 4: Record the result.

Step 5: Power off the pump.

Step 6: Repeat the 2–5 steps 3 times for each speed.

Step 7: Replace another impeller to test again.

**Figure 7:**
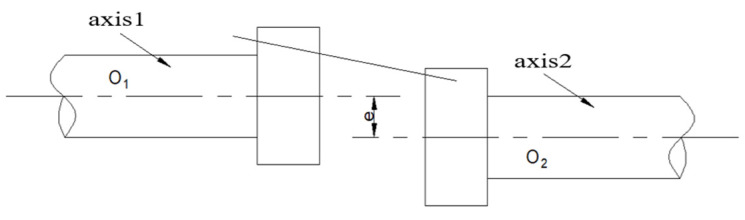
Parallel misalignment.

**Figure 8:**
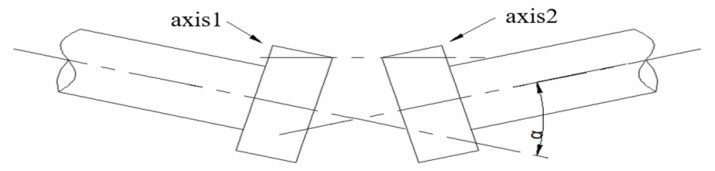
Angle misalignment.

(2) Misalignment fault:

The misalignment includes parallel misalignment and angle misalignment, Figure 7 and Figure 8 show the actual condition.

Step 1: Adjust the pump to work in the best condition.

Step 2: Adjust the parallel misalignment as the following level shown in Table 3.

Under the same parameters of parallel misalignment or angle misalignment, three different speeds of 1800, 2400, and 3000 r/min were run for 5–10 min.

**Figure 9:**
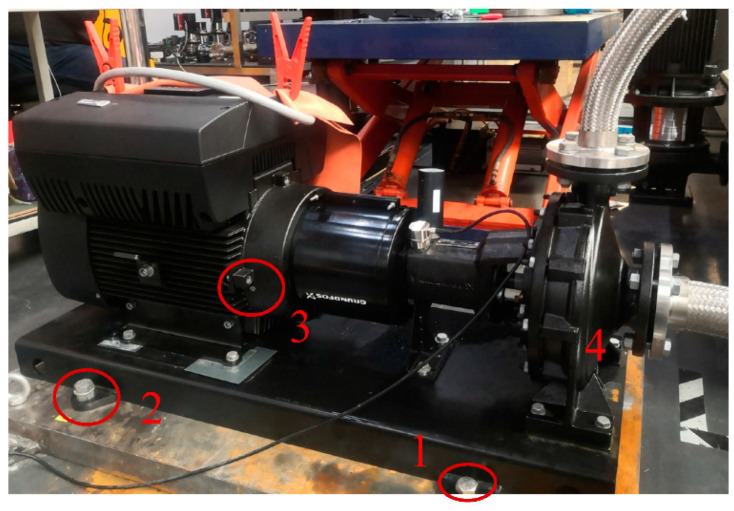
Four corner screws in the pump. 1, 2, 3 and 4 represent the screws on the four corners of the pump foundation.

(3) Loose fault:

As shown in Figure 9, the four corner screws were defined as 1, 2, 3, and 4. They were tightened before the test. During the test, the screws were loosened one by one, and the screw status and cloud results were recorded (every change should be kept for 5–10 min to make sure the loosening changed the pump state).

The pump speed was set to 1800, 2400, or 3000 r/min.

(4) Bearing fault:

Housings for bearings in the centrifugal pump were artificially worn to different degrees, and the worn parameters of the experiments were set as shown in Table 4.

To test it, follow these steps:

Step 1: Install one worn bearing on the pump.

Step 2: Set the pump speed to 1800, 2400, or 3000 r/min.

Step 3: Power on the pump, and keep the pump working for 5–10 min.

Step 4: Record the result.

Step 5: Power off the pump.

Step 6: Repeat the 3–5 steps 3 times for each speed.

Step 7: Replace another bearing to test again.

### 5.3. Test Results and Analysis

Statistical calculation methods for misdiagnosis, missed diagnosis, and correct diagnosis in the test: (1) Monitor the vibration amplitude of the sensor in three axial directions and the fault indication index value simultaneously. (2) If the total vibration value in either direction of the sensor exceeds the set alarm threshold, there must be a diagnostic result. If there is no diagnostic result output, it belongs to missed diagnosis. If the diagnosis result is inconsistent with the actual fault, it is a misdiagnosis. (3) If the total vibration value in either direction of the sensor does not exceed the set alarm value, diagnosis can be omitted. (4) The total vibration value does not exceed the alarm threshold, but a certain characteristic index change triggers the diagnostic logic. If a diagnosis is made, when the diagnostic output result is consistent with the actual form of failure, the diagnosis is considered correct; if the output diagnostic results do not match the actual situation, it is a misdiagnosis. (5) Use precision (P), recall (R), error rate (ER), and accuracy (A) [36,37] as performance indicators to evaluate the fault diagnosis results. P, R, ER, and A can be obtained using the following equations:

(1) $$P=\frac{\mathrm{TP}}{\mathrm{TP}+\mathrm{FP}}$$

(2) $$R=\frac{\mathrm{TP}}{\mathrm{TP}+\mathrm{FN}}$$

(3) $$\mathrm{ER}=\frac{\mathrm{FP}+\mathrm{FN}}{\mathrm{TP}+\mathrm{TN}+\mathrm{FN}+\mathrm{FP}}$$

(4) $$A=\frac{\mathrm{TP}+\mathrm{TN}}{\mathrm{TP}+\mathrm{TN}+\mathrm{FN}+\mathrm{FP}}=1-\mathrm{ER}$$

In Equations (1)–(4), the terms TP, FN, FP, and TN, respectively, represent true positive, false negative, false positive, and true negative, as shown in Table 5. In a practical test statistic, the value of TP is the number of correct diagnoses, the value of FP is the number of misdiagnoses, and the value of FN is the number of missed diagnosis. The sum of TP, TN, FN, and FP is the number of test samples.

According to the above principles, the imbalance fault and misalignment fault were tested and recorded at three speeds of 1800, 2400, and 3000 rpm, respectively, and the statistical results were as follows.

As shown in Table 6, for the imbalance fault, the diagnostic accuracy and precision of the wired sensor are slightly lower than that of the wireless sensor, but the recall rate of the diagnosis is higher than that of the wireless sensor. For misalignment faults, the diagnostic accuracy, precision, and recall of wired sensors are higher than those of wireless sensors. Because in practical applications, to meet the power consumption requirements of the battery, wireless sensors often use a data collection and upload cycle of 30 min or even longer. Compared with wired sensors, the collection and upload interval are longer, which means that it is easy to cause misses or misdiagnoses. The wired way can continuously sample and transmit to ensure the accuracy of data, and the feature calculation can be placed on the edge device, which can effectively reduce the requirements for network bandwidth and improve real-time monitoring.

We tested the faults such as loose fault, bearing fault, cavitation, etc., in the same method, and the automatic diagnosis accuracy, precision, and recall obtained by the test are shown in Table 7. In Table 7, for bearing fault and cavitation fault, the shutdown operation was carried out due to the short duration after the failure occurred, and the acquisition and transmission mechanism of the wireless sensor had the possibility of not collecting data, so the test results of the wireless sensor were not counted. However, tests have also shown that if fault data is collected through wireless sensors during equipment failure, correct fault classification can be performed.

The experimental test results show that the designed sensors and the sensor-based centrifugal pump monitoring and diagnosis system can effectively collect the dynamic vibration data of the equipment during operation. The fault features are extracted in the software system and the threshold self-learning of the feature indicators is completed. The diagnosis system can make correct judgments on faults such as loose faults, impeller imbalance, rotor misalignment, bearing wear, and cavitation. For wired sensors overall, the diagnostic accuracy is over 85.71%, the diagnostic precision is more than 90.20%, and the diagnostic recall rate exceeds 85.71%. For wireless sensors overall, the diagnostic accuracy is over 80.33%, the diagnostic precision is more than 90.70%, and the diagnostic recall exceeds 83.13%.

In addition, except for imbalance fault, the acquisition methods of wired sensors and collectors have higher diagnostic accuracy, precision, and recall than wireless sensors in various faults. Due to the limitations of its battery and transmission mechanism, wireless sensors cannot continuously collect signals in real time, resulting in an inability to effectively monitor and judge some occasional and sudden faults, resulting in lower performance. However, the wireless sensor method is still effective for faults that deteriorate over time. It can be used as an effective and low-cost method for monitoring the operation status of equipment, and at the same time, the installation is more convenient than the wired method.

## 6. Conclusions

Taking centrifugal pumps as the object of action, this paper studied and designed digital devices such as wireless sensors, wired sensors, and collectors, and centrifugal pump monitoring and a diagnosis IoT system that uses these digital devices. Targeted tests and verifications were carried out for common failure forms. The test results show that the designed sensors and sensor application system can make good use of the centrifugal pump fault mechanism and automatically diagnose the equipment failure. Using the wired sensor and collector, the diagnostic accuracy rate is more than 85%. As a low-cost, easy-to-implement solution, wireless sensors can also monitor progressive faults very well.

The research of the paper provides a feasible IoT system and a set of practical tools for equipment health management and digital application of industrial enterprises. We will also further improve the condition monitoring and fault diagnosis system of centrifugal pumps in future work.

With the deepening of the application, there will be an increasing amount of sensor monitoring data connected to the server. These data can be used on the server for the learning and optimization of the fault diagnosis mechanism model, to improve the threshold judgment standard of the fault characteristic data, and to improve the diagnostic accuracy of the existing methods. On the other hand, it can also be used to design a hybrid intelligent diagnosis model driven by data and mechanism in the future to promote the application of digital intelligence.

## Figures and Tables

**Table 1:** The types of faults that often occur when centrifugal pumps are running.

Common faults in the running state of centrifugal pumps	Mechanical failure	Imbalance fault
		Misalignment fault
		Loose fault
		Bearing fault
	Fluid failure	Cavitation
		Water hammer
		Abnormal flow passage

**Table 2:** Main parameters of the experimental pump.

Parameter	Numeric Value
Type of pump	NKE 40-250/255
Rated power (kW)	22
Rated speed (rpm)	2940
Center height (mm)	185
Number of impeller blades	5
Mains frequency (Hz)	50
Type of bearing	Cylindrical roller bearing

**Table 3:** Parameter setting of misalignment fault.

Serial Number	Misaligned (Parallel) Level (μm)	Misaligned (Angle) Level (μm)
1	Initial	Initial
2	−500	−3000
3	−200	−1500
4	+200	+3000
5	+500	+1500

**Table 4:** Parameter setting of bearing outer ring wear amounts.

Serial Number	X (Length) (mm)	Y (Width) (mm)	Z (Depth) (mm)
1	0	0	0
2	3	15	1
3	6	15	1
4	15	15	1

**Table 5:** The status table of real value and diagnosis.

	Diagnosis: Fault A	Diagnosis: Normal or Other Faults
Real: Fault A	True positive	False negative
Real: Normal	False positive	True negative

**Table 6:** Statistical records of tests for imbalance fault and misalignment fault.

Type of Fault	Type of Sensor	Number of Test Samples	Sample of Normal	Sample of Failure	Number of Correct Diagnosis	Number of Missed Diagnosis	Number of Misdiagnosis	Diagnostic Accuracy	Diagnostic Precision	Diagnostic Recall
Imbalance fault	Wired	90	35	55	46	4	5	90.00%	90.20%	92.00%
	Wireless	88	41	47	39	4	4	90.91%	90.70%	90.70%
Misalignment fault	Wired	38	20	18	17	0	1	97.37%	94.44%	100%
	Wireless	37	23	14	11	2	1	91.89%	91.67%	84.62%

**Table 7:** Experimental test results.

The Type of Fault	The Type of Sensor	Diagnostic Accuracy	Diagnostic Precision	Diagnostic Recall
Imbalance fault	Wired	90.00%	90.20%	92.00%
	Wireless	90.91%	90.70%	90.70%
Misalignment fault	Wired	97.37%	94.44%	100%
	Wireless	91.89%	91.67%	84.62%
Loose fault	Wired	95.08%	100%	91.43%
	Wireless	80.33%	91.30%	83.13%
Bearing fault	Wired	100%	100%	100%
	Wireless	N.A.	N.A.	N.A.
Cavitation fault	Wired	85.71%	100%	85.71%
	Wireless	N.A.	N.A.	N.A.

## Data Availability

The data presented in this study are available on request from the corresponding author. The data are not publicly available due to privacy or ethical restrictions.
